# Validation of the Swedish Quality Register for Ear Surgery – SwedEar

**DOI:** 10.1186/s12911-023-02340-y

**Published:** 2023-10-26

**Authors:** Malin Berglund, Sara Olaison, Eva Westman, P. O. Eriksson, Lena Steger, Åsa Bonnard

**Affiliations:** 1 Department of Otorhinolaryngology, NU Hospital Group, Trollhättan, Sweden; 2 Department of Biomaterials, Institute of Clinical Sciences, Sahlgrenska Academy, University of Gothenburg, Gothenburg, Sweden; 3 Department of Otorhinolaryngology, Örebro University Hospital, Örebro, Sweden; 4 Faculty of Medicine and Health, Örebro University, Örebro, Sweden; 5 Department of Clinical Sciences, Otorhinolaryngology, Umeå University, Site Sundsvall, Umeå, Sweden; 6 Medical Unit of Ear, Nose and Throat, Hearing and Balance, Karolinska University Hospital, Stockholm, Sweden; 7 Department of Surgical Sciences, Otorhinolaryngology, Uppsala University Hospital, Uppsala, Sweden; 8 Department of Otorhinolaryngology, Gävle Hospital, Gävle, Sweden; 9 Division of CLINTEC, Department of Otorhinolaryngology, Karolinska Institutet, Stockholm, Sweden

**Keywords:** Ear surgery, Myringoplasty, Chronic otitis, Validation, Quality register, Health quality improvement

## Abstract

**Background:**

The Swedish Quality Register for Ear Surgery (SwedEar) is a national register monitoring surgical procedures and outcomes of ear surgery to facilitate quality improvement. The value of the register is dependent on the quality of its data. SwedEar has never been validated regarding data quality or missing entries. Therefor, the purpose of this study was to assess coverage, completeness and response rate in the register and validate the physicians’ reported data accuracy.

**Methods:**

In this validation study, the completeness, response rate and missing registrations were analysed. Data in SwedEar were compared with the yearly collected statistics of otosurgical procedures in The Swedish Otosurgical Society and the comparison of rates between groups was calculated with Fisher’s exact test. Validation of registered data accuracy was performed on every 20^th^ registered case during a five-year period. Data were reabstracted from medical records and compared with the original registration. Interrater agreement, reliability measures, Cohen’s kappa, Gwet’s AC1 and positive predictive value were calculated.

**Results:**

SwedEar has a coverage of 100%. The completeness of registered cases was 84% and the response rate was 74%. The validation of data accuracy assessed 13 530 variables, including audiograms. Less than 3% of incorrect or missing variables were identified. For most of the pre- and postoperative variables the Kappa and Gwet´s AC1 results show an almost perfect agreement (> 0.80). For audiogram data the ICC shows an excellent reliability (> 0.9) for all but one value.

**Conclusion:**

This validation shows that SwedEar has excellent coverage, high completeness, and that the data in the register have almost perfect reliability. The data are suitable for both clinical and research purposes. Further efforts to improve completeness are warranted.

## Background

National registers, monitoring the outcome and quality of healthcare, are increasing in numbers in several countries, especially in the European Union and the United States [1–5]. In Sweden almost 100 government-maintained nationwide quality registers have been developed, and four registers concerning ear surgery exist [6]. The ear surgery registers collect data regarding chronic ear surgery, otosclerosis surgery and cochlear implantation in children and adults. The primary aim of the Swedish registers is quality control by providing comparative data, but the data collected can also be used for research [7, 8]. Internationally, population-based quality registers for chronic ear surgery are sparse. An attempt to establish an international register for ear surgery was made by the European Otology Database Project Group resulting in the Common Otology Audit started in 2004, with some early results reported [9].

The Swedish Quality Register for Ear Surgery (SwedEar) (former Quality Register for Myringo- and Ossiculoplasty (QRMO)) is a national register for ear surgery. It was founded in 1997 for registration of myringoplasties in noninfected ears. After revision in 2013, the register included all conventional myringoplasties, fat graft myringoplasties, and ossiculoplasties. Cholesteatoma and retraction pockets were excluded. The latest expansion was in October 2020, where all chronic ear surgeries including cholesteatoma were included [8].

In Sweden, register data have been increasingly used for quality assessment and research. The Swedish governmentally ruled health system and personal identification numbers (PINs) which enable longitudinal follow-up, provide unique opportunities for epidemiological research. Universal, tax-supported healthcare for all citizens is provided by the Swedish National Healthcare service. The Swedish population has, in general, a high confidence in the government, which makes patients willing to participate in registers. In the quality register there is no need for written consent because of an opt-out system. These factors contribute to high completeness in registered data. However, participation in the quality registers is voluntary for the surgical units, and a low confidence in the register and its results, lack of time or forgetfulness can contribute to lower coverage.

The value of a register is dependent on the quality of its data why the knowledge of data validity is important. This includes coverage of participating surgical units, completeness of registered procedures and completeness and validation of registered variables [10, 11]. Different methods can be used, for example by comparing the data to an external register or reabstraction of medical records [5].

Since 1987, all otosurgical units in Sweden have reported the number and type of procedures to The Swedish Otosurgical Society, SÖF, and these data are regarded here as the gold standard [12]. The patients are reported on a group level, and this can be used to estimate completeness.

SwedEar has never been validated regarding data quality or missing entries. Therefore, the aim of this study was to validate the Swedish Quality Register for Ear Surgery, SwedEar. The results are presented for coverage, completeness, response rate, accuracy of diagnosis and type of intervention and other registered data. Furthermore, to control selection bias, the results of registered patients were analysed and compared with the results of patients with incomplete or missing data.

## Methods

This is a validation study of the data in SwedEar, a national quality register for ear surgery. Data were retrospectively compared in two separate ways; one using five surgical units chosen to represent Swedish national healthcare and the other using a randomly selected subcohort from the register. Further details regarding each study are described below.

### Data in SwedEar

The medical data in SwedEar is collected per- and one year postoperatively and includes age, gender, surgical unit, indication for surgery, surgery type and techniques, preoperative infection and antibiotic treatment, preoperative hearing and postoperative results as hearing results and complications. On a voluntary basis, the surgeon can add a pseudonym to be able to follow his or her results. A patient-reported outcome measure (PROM) is distributed to the patient approximately one year after surgery. The PROM was not included in the validation. Participation in the register is voluntary, but all 33 units performing ear surgery in Sweden are included, giving 100% coverage. A 100% completeness is not possible to obtain due to a need for a PIN in order to be registered in SwedEar. This excludes individuals without a residence permit in Sweden. The completeness in this article will therefore be calculated as an adjusted completeness.

### Data collection and calculation of completeness of cases, response rate, and healing rate in individuals with incomplete or missing data

Five units, three university hospital units and two county hospital units, were selected for validation of completeness, response rate and control of incomplete and missing data for all surgeries reported to SwedEar during the year 2017.

For validation of completeness, the data were compared with the yearly collected statistics of otosurgical procedures from SÖF. The number of surgeries missing in SwedEar (in comparison with the numbers from SÖF) was compared with data stored in the surgical planning software and medical records at each unit to ensure the correctness of the diagnosis. Data were collected regarding all myringo- and ossiculoplasties missing in SwedEar including individuals not possible to register (lack of PIN).

To analyse the frequency of tympanic membrane (TM) healing rate in cases with incomplete or missing data, medical records were examined for all individuals with a lack of a registered postoperative form or not included in SwedEar. The recommended follow-up was at one year postoperatively, but if this was not obtained, the latest date following surgery was accepted (minimum 4 weeks). The healing rate was then compared with that of the registered individuals.

### Data collection for the validation of registered data accuracy and completeness.

Between October 2013 and October 2018, 4593 surgeries were registered, of which 2479 had both pre- and postoperative data. Due to the great number of cases in the register, the validation process was performed on every 20^th^ registered case (*n* = 124), representing 26 out of 33 reporting units. This refers to a random selection process. All units were contacted for reabstraction of the digital medical records for all registered data included in the two pre- and postoperative forms. The reabstracted data were collected and thereafter compared with the original data with the possible results of “correct”, “incorrect” or “missing”. The variables examined, included a total of 110 possible data points per ear surgery (medical pre- and postoperative form).

The composition of pre- and perioperative data in SwedEar in total, the subgroup with both pre- and postoperative data (follow-up) and the validated group was reasonably comparable (Table 1). The percentage of women as well as ears not exhibiting infection at surgery was higher in the validated group, but there was no statistically significant difference.

**Table 1:** Comparison of preoperative data from SwedEar

	SwedEar All	SwedEar Pre- & postop	Validated	*p*
N	4593	2479	124	
Age (mean, y)	32	32	33	
Sex (women)	2350 (51%)	1263 (51%)	77 (62%)	0.051
Right ear	2373 (52%)	1253 (51%)	59 (48%)	0.48
Primary ear surgery	1176 (26%)	623 (25%)	26 (21%)	0.47
General anaesthesia	4431 (96%)	2387 (96%)	118 (95%)	0.70
Indication infection free	3861 (84%)	2091 (84%)	107 (86%)	0.51
Indication hearing	2695 (59%)	1488 (60%)	77 (62%)	0.39
Myringoplasty performed	4337 (94%)	2355 (95%)	119 (96%)	0.46
Myringoplasty by plugging	435 (9%)	220 (9%)	17 (14%)	0.17
Ossiculoplasty	929 (20%)	494 (20%)	22 (18%)	0.52
Preoperative antibiotics	271 (6%)	150 (6%)	7 (6%)	0.96
Infection free at surgery	4055 (88%)	2210 (89%)	117 (94%)	0.08
Perioperative antibiotics	842 (18%)	471 (19%)	23 (19%)	0.80

SwedEar All = all surgeries registered in the register from the start in October 2013 until the time of validation; SwedEar Pre- & postop = surgeries in SwedEar with a registered follow-up; and validated = validated surgeries. *P* = Chi2 test for dichotomous values and ANOVA for continuous values. *Y* years

### Statistical analysis

The distribution of variables is presented as the number and percentage for dichotomous values and as the mean and SD for continuous values. Coverage was calculated as the percentage of units participating in the register and completeness of cases as procedures registered in the register from the selected units compared with SÖF statistics. Adjusted completeness was calculated on the subgroup of individuals having a PIN. The response rate was calculated as the percentage of individuals with a registered postoperative follow-up. The difference in results between registered and missing individuals was calculated with Fisher’s exact test.

For validation of data accuracy and completeness, the dichotomous values are presented as the percentages of agreement between registered data and reabstracted data calculated with observed agreement. To illuminate possible problems related to prevalence, both Cohen’s Kappa and Ghwet’s AC1 with 95% CI were used [13]. Kappa coefficients can be simplified into 5 categories. Values ≤ 0.20 indicated slight agreement, 0.21–0.40 indicated fair agreement,0.41–0.60 indicated moderate agreement, 0.61–0.80 indicated substantial agreement, and values above 0.80 indicated almost perfect agreement [14, 15]. The positive predictive value (PPV) is calculated to illustrate the validity of data correctness. For validation of continuous values, limits of agreements and intraclass correlation coefficient (ICC) were used [16, 17]. For analyses of systematic differences, the sign test was used for dichotomous variables, and the Wilcoxon signed rank test for continuous variables. All significance tests were two-sided and conducted at the 5% significance level.

Data gathering from medical records was performed in Excel. IBM SPSS Statistics 28.0.0.0 and SAS software 9.4 (maintenance release: 9.04.01M6P111518) were used for data analyses and calculations.

## Results

### Analysis of coverage, completeness of cases, and response rate

All ENT units performing ear surgery participate in the register, resulting in a coverage of 100%. The five units selected for validation covered 35.9% (*n* = 371) of all 1031 procedures reported to SÖF in 2017 (range 24.7–37.8% regarding type of surgery, Table 2).

**Table 2:** Number of surgeries presented for the different types of surgeries included in the present study

Type of surgery	Total N (%)	Unit 1	Unit 2	Unit 3	Unit 4	Unit 5
Conventional myringoplasty Fat graft myringoplasty	263 (37.8) 19 (24.7)	39 3	18 4	33 1	10 8	163 3
Myringo-ossiculoplasty	50 (33.1)	1	9	7	2	29
Ossiculoplasty	40 (37.0)	3	3	8	2	26
All surgeries	372 (35.9)	46	34	49	22	221

The percentage of the total number of surgeries, in total and by unit, is in regard to reported statistics to The Swedish Otosurgical Society

Fourteen individuals who underwent surgery, did not have a PIN (3.8%), which is why 357 out of 371 surgeries would have been possible to include in SwedEar. Of these, 300 individuals were included, giving an adjusted completeness of 84.0%. The total completeness, including individuals lacking PIN is 80.9%. Of all registered cases, 224 had a return visit resulting in a response rate of 74.7% (Table 3).

**Table 3:** Ear surgeries, follow up and tympanic membrane healing rate reported to the Swedish Quality Register for Ear Surgery (SwedEar) in 2017

	N (%)	Range %	Missing N
Adjusted completeness of cases	300/357 (84.0)	68.2–100	
Response rate (registered follow-up)	224/300 (74.7)	66.7–100	
Healing rate (in register, with follow-up)	206/224 (92.0)	70.6–100	
Healing rate (in register, no registered follow-up)	62/73 (84.9)	66.7–90.0	3
Healing rate (not registered)	51/62 (82.3)	54.5–92.7	10

The number of ear surgeries identified in the surgical planning systems with a personal identity number (PIN) was 357 out of 371. In SwedEar, 300 procedures were registered. The adjusted completeness was calculated for patients with PINs. Range = the range of percentages for the five units. Missing = patients with no postoperative control > 4 weeks were excluded from the calculation of the healing rate

### Analysis of the tympanic membrane healing rate in all subgroups

The TM healing rate was 92.0% in the cases with a registered follow-up (*n* = 224) (Table 3). Out of 76 cases without a follow-up, three were lost to follow-up (3.9%). In the remaining 73 cases the TM healing rate was 84.9%. Among the 72 cases not registered in SwedEar, medical records were found in 62 cases showing a healing rate of 82.3%. The nonregistered group includes individuals lacking PIN. There was a significant difference in the healing rate between the group with a registered follow-up and the nonregistered group (*p* = 0.032) but not the registered group lacking a follow-up (*p* = 0.11).

### Completeness and accuracy of diagnosis, type of intervention and registered data

**Fig. 1:**
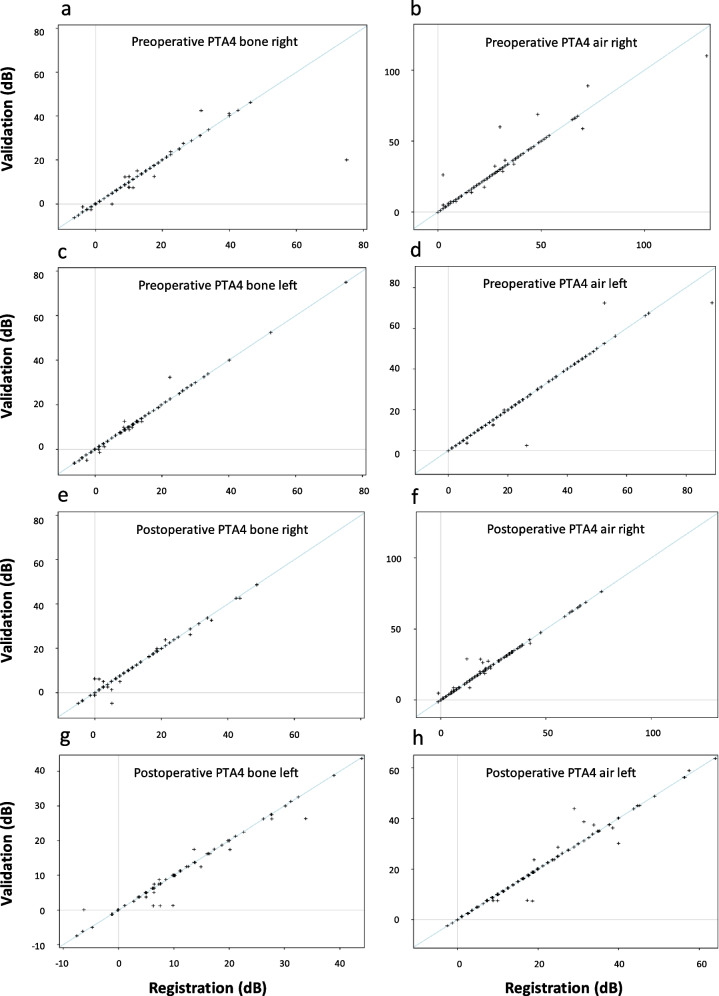
Scatterplots representing pre- and postoperative PTA4 for air and bone as well as the right and left ears. Values in SwedEar (registration) on the x-axis and validated values (validation) on the y-axis. All measures are presented in decibels (dB)

A total of 123 out of 124 cases could be validated, resulting in 13,530 data points. In total, 395 incorrect or missing variables (2,9%) were identified. Missing values accounted for 10% (*n* = 41, 0.3% of total data), giving a completeness of data of 97.7%. The incorrect or missing values were mostly identified within audiogram registrations (*n* = 178, 45%), but all different sections were represented. Eight percent of the values (*n* = 33) could not be controlled due to uncertainty in the interpretation of medical records. Regarding the error in audiogram registrations, the difference in pure tone average between original and corrected values was approximately 1 dB for air conduction and 2 dB for bone conduction at the group level (Table 4). Scatterplots of the original and validated values are presented in Fig. 1. Severe errors occurred in 6.8% of all errors (*n* = 27, 0.2% of all data points) and were represented by incorrect side-indication, surgery, main outcome measure (healed TM and postoperative infection) and audiogram (wrongly indicated deaf ear or side mix-up).

**Table 4:** Interrater agreements are presented as the limits of agreement, ICC and equal registration values regarding audiogram data

		N	Mean Difference	Limits of agreement	Systemat Difference^a^	ICC	Equal registration values		PTA4 difference
		(%)		(95%)	*p* value	CI (95%)	0 (%)	< = 5 (%)	(mean dB)
*Preoperative PTA4*									
Right ear	Bone	86 (92)	-0.54	-12.64–11.56	0.85	0.87 (0.81–0.91)	81.4	97.7	-1.24
	Air	123 (100)	0.46	-8.85–9.76	0.54	0.97 (0.95–0.98)	86.2	95.1	0.76
Left ear	Bone	81 (90)	0.05	-2.51–2.60	0.62	0.99 (0.99–1.00)	87.7	98.8	-2.04
	Air	119 (97)	-0.22	-6.59–6.14	0.24	0.98 (0.97–0.99)	93.3	97.5	-0.72
*Postoperative PTA4*									
Right ear	Bone	81 (93)	-0.09	-3.30–3.13	0.68	0.99 (0.98–0.99)	82.7	97.5	-0.54
	Air	122 (100)	0.32	-3.69–4.32	0.12	0.99 (0.99–0.99)	89.3	96.7	-0.43
Left ear	Bone	79 (92)	-0.30	-3.90–3.30	0.15	0.98 (0.98–0.99)	83.5	94.9	-0.41
	Air	117 (95)	-0.01	-4.74–4,72	0.98	0.99 (0.98–0.99)	89.7	95.7	-0.19

Bone conduction on both sides was not always performed. ^a^The Wilcoxon signed rank was used to test the difference. N = cases validated with percentage (%) of all cases with at least one observation at registration or validation, *CI* Confidence Interval, *ICC* Intraclass Correlation Coefficient. The PTA4 difference represents the change in mean dB for all cases between the reported and validated values

In Table 5, the results of observed agreement, Kappa, Gwet’s AC1 and PPV (positive predictive value) are presented for the most important variables. The observed agreement was more than 0.94 for all variables. The Kappa and Gwet’s AC1 results show an almost perfect agreement (> 0.80) in all except four variables. For these values, only the prevalence-insensitive Gwet’s AC1 showed an almost perfect agreement. There were no incorrectly registered cases regarding the inclusion criteria (diagnosis and the two interventions, myringoplasty and ossiculoplasty), giving a PPV of 100% (*n* = 123). All except one variable had a PPV of 82% or higher, indicating very good validity. The variable with a lower PPV, “preoperative antibiotics”, had very few positive outcomes. Seven cases were originally classified as positive values, but 3 were judged as false positives in the validation, giving a PPV of 0.57.

**Table 5:** Analyses of agreement between pre- and postoperative data originally registered in SwedEar and at validation

Variable	Total N	Match N (%)	SwedEar yes Val yes N (%)	SwedEar no Val no N (%)	Non Match N (%)	SwedEar yes Val no N (%)	SwedEar no Val yes N (%)	Observed agreement CI (95%)	Cohen’s kappa CI (95%)	Gwet AC1 CI (95%)	PPV CI (95%)	Systematic difference *p* value^*^
*Preoperative form*												
Operated right side	123	122 (99.2)	65 (52.8)	57 (46.3)	1 (0.8)	1 (0,8)	0 (0)	0.99 (0.96–1.00)	0.98 (0.95–1.00)	0.98 (0.95–1.00)	1.00 (1.00–1.00)	0.317
Prior ear surgery	122	120 (98.4)	26 (21.3)	94 (77.0)	2 (1.6)	0 (0)	2 (1.6)	0.98 (0.94–1.00)	0.95 (0.89–1.00)	0.97 (0.94–1.00)	1.00 (1.00–1.00)	0.157
Myringo performed	123	123 (100)	118 (95.9)	5 (4.1)	0 (0)	0 (0)	0 (0)	1.00 (0.97–1.00)	1.00 (1.00–1.00)	1.00 (1.00–1.00)	1.00 (1.00–1.00)	-
Ossiculoplasty performed	123	121 (98.4)	22 (18.0)	99 (80.4)	2 (1.6)	0 (0)	2 (1.6)	0.98 (0.94–1.00)	0.95 (0.87–1.00)	0.98 (0.94–1.00)	1.00 (1.00–1.00)	0.157
Preoperative antibiotics	122	118 (96.7)	4 (3.3)	114 (93.4)	4 (3.3)	3 (2.5)	1 (0.8)	0.97 (0.92–0.99)	0.65 (0.33–0.97)	0.96 (0.93–1.00)	0.57 (0.20–0.94)	0.317
Infection free at surgery	121	120 (99.2)	115 (95.0)	5 (4.1)	1 (0.8)	1 (0.8)	0 (0)	0.99 (0.95–1.00)	0.90 (0.72–1.00)	0.99 (0.97–1.00)	0.99 (0.97–1.00)	0.317
Perioperative antibiotics	120	119 (99.2)	21 (17.5)	98 (81.7)	1 (0.8)	1 (0.8)	0 (0)	0.99 (0.95–1.00)	0.97 (0.92–1.00)	0.99 (0.96–1.00)	0.95 (0.87–1.00)	0.317
Postoperative antibiotics	120	117 (97.5)	14 (11.7)	103 (85.8)	3 (2.5)	2 (1.7)	1 (0.8)	0.98 (0.93–0.99)	0.89 (0.77–1.00)	0.97 (0.93–1.00)	0.88 (0.71–1.00)	0.564
*Postoperative form*												
Intact ear drum	122	120 (98.4)	98 (80.3)	22 (18.0)	2 (1.6)	1 (0.8)	1 (0.8)	0.98 (0.94–1.00)	0.95 (0.87–1.00)	0.98 (0.94–1.00)	0.99 (0.97–1.00)	1.000
Infection free ear	123	120 (97.6)	118 (95.9)	2 (1.6)	3 (2.4)	2 (1.6)	1 (0.8)	0.98 (0.93–0.99)	0.56 (0.11–1.00)	0.97 (0.94–1.00)	0.98 (0.96–1.00)	0.564
Dry ear	122	121 (99.2)	115 (94.3)	6 (5.0)	1 (0.8)	0 (0)	1 (0.8)	0.99 (0.96–1.00)	0.92 (0.76–1.00)	0.99 (0.97–1.00)	1.00 (1.00–1.00)	0.317
Ear drum no deep retraction	123	118 (95.9)	114 (92.7)	4 (3.3)	5 (4.1)	1 (0.8)	4 (3.3)	0.96 (0.91–0.99)	0.60 (0.27–0.92)	0.95 (0.91–1.00)	0.99 (0.97–1.00)	0.180
Postoperative infection	122	116 (95.1)	9 (7.4)	107 (87.7)	6 (4.9)	2 (1.6)	4 (3.3)	0.95 (0.90–0.98)	0.72 (0.51–0.93)	0.94 (0.89–0.99)	0.82 (0.59–1.00)	0.414
Planned additional surgery	112	110 (98.2)	14 (12.5)	96 (85.7)	2 (0.2)	1 (0.9)	1 (0.9)	0.98 (0.94–1.00)	0.92 (0.82–1.00)	0.98 (0.94–1.00)	0.93 (0.81–1.00)	1.000

Interrater agreements are represented as observed agreement, Cohen’s kappa and Gwet’s AC1 for pre and postoperative variables. The positive predictive value (PPV) represents the validity of data correctness. SwedEar = registered data in SwedEar. *Val* validated data, *N* number of cases, *CI* Confidence Interval

^*^Systematic difference, *p* values, McNemar’s test. The McNemar’s test uses only information from patients in the non-match group

Regarding the validation of the audiogram, the results of limits of agreements, interclass correlation coefficient (ICC) and equal registration values for 0 and <  = 5% are presented in Table 4. Not all PTA4 values could be evaluated due to missing values in either the register or validation, and 92–100% were included in the calculations. In general, the limits of agreement are low, with an interval range of ± 3–12 dB, predominantly greater in the preoperative measurements. The ICC showed excellent reliability (> 0.9) for all values except the preoperative PTA4 bone right, which had good reliability (0.87). Over 81.4% of all PTA4 values are equally registered in the register and the validation, and > 94.9% are within a 5 dB difference.

## Discussion

Data completeness and validity are of major importance for registers, as they are essential for reliable conclusions from the registered data. The validity of the data in SwedEar has not been assessed before. This validation study shows that the data are of high quality and have excellent reliability. The coverage was 100%, the adjusted completeness of cases was 84.0% and the completeness of registered data was 97.7%. The registered data show an almost perfect agreement (Gwet’s AC1 > 0.80) for all and a PPV over 88% for all but one of the dichotomous variables and excellent reliability (ICC > 0.9) for all but one of the audiogram values. The accuracy of diagnosis and type of interventions all had a PPV of 100%. These results imply that SwedEar can be used for monitoring quality aspects and results in myringo- and ossiculoplasty for clinical, research and public purposes.

Validation of registers can be performed in different ways. Many registers, such as SwedEar, assess the completeness of cases by cross-linkage to another register [18–20], and others use reabstraction of the digital medical records for all cases during a particular period [21, 22]. The latter is also often used for the validity of data accuracy [20, 23, 24]. This study used surgery data from SÖF as a comparison for validation. Most registers use the National Patient Register from The Swedish National Board of Health and Welfare. It contains statistics regarding all diagnoses and surgical procedures in Sweden and has been mandatory for all regions since 1987 [25, 26]. This register is generally regarded as the gold standard for comparison in Sweden. Unfortunately, the classification of surgical procedures in otosurgery is not surgery-type specific, which is why the code for myringoplasty and ossiculoplasty may be used in other types of surgeries not included in SwedEar. In addition, it often lacks side indications (right or left ear) for surgery.

Completeness in a register is linked to the term sensitivity (the number of true cases in the register divided by the actual number of true cases). SwedEar has perfect coverage and high completeness of cases, but not as high as some other quality registers in Sweden. For example, registers concerning fractures, breast cancer, and neonatal care have reported completeness of over 90% [19, 20, 26]. Calculating completeness by cross-linking to another register could imply that some missing or wrongly included cases in one of the registers will never be detected. Since SÖF only registers on a group level, individual comparisons are not possible. This affects the certainty of completeness. A 100% completeness could never be attained in SwedEar due to register constraints, and individuals lacking PINs cannot be included. To calculate the completeness only for patients with PINs, a subgroup of five units was used for in-depth analysis. Our results are therefore presented as an adjusted completeness. The Swedish Association of Local Authorities and Regions has established criteria for levels of certification of the national quality registers in a four-grade scale, ranging from a candidacy register to a level one register [27]. To reach the level one certification criteria, the coverage and completeness have to be higher than 85%. More work has to be done to improve the completeness of SwedEar. Since this study, enlargement of the register with the inclusion of all chronic ear surgeries was conducted to reduce the loss of registrations due to the uncertainty of inclusion criteria. The results have also been presented at several SÖF-meetings and in the journal of the Swedish Otorhinolaryngology, Head and Neck Surgery Society, to inspire improvement on an individual level. Increased direct feedback to each unit is planned.

In general, the validity of SwedEar is good with a low frequency of errors, 2.9%, and an almost perfect coherence of data. This can be compared with the 5.9% errors in the Swedish National Tonsil Surgery register [22] and a mean of 9.8% from 42 articles of primary register data presented by Nahm et al. 2008 [28]. On the other hand, Kirch et al. suggested an acceptable database error rate under 1% [29]. Ours is slightly higher but much lower than in many comparable registers and with no systematic errors. Most of the errors occurred in the audiograms and on a group level the small difference of 1–2 dB is of no clinical significance. However, effects in subgroups cannot be ruled out. Today all values are inserted manually in the register and a system has been introduced with warnings for illogical values. A future automatic procedure for audiogram input could further improve the quality of audiogram data.

The only value with a low PPV was “preoperative antibiotics”. The reason for this might be the difficulty of finding information from different sources of medical records, and the “preoperative” time frame is a question of interpretation. Some of the variables, for example, “infection free ear” and “eardrum no deep retraction”, could be a question of the subjectivity of the status of the ear between the treating surgeon initially registering the case and the validator. These types of questions are assessed to be difficult to comprehend, and in the renewed register from 2020, the questions are rephrased and/or an explanation is added to facilitate understanding.

The TM healing rate reported in other studies varies between 65–92% [30–33]. The findings in the present study show an overall good TM healing rate for all groups, in accordance with international results. There was, however, a significantly lower TM healing rate among nonregistered cases, 82 vs. 92%. The explanation for this difference is not fully understood. In the group of cases lacking a registered return visit, there was no significant difference in TM healing results. This might imply that a bad result was not the reason for failing registration.

### Limitations of the study

In this study, there is a risk of errors due to manual entries of the validation data. To minimize this risk, all individuals involved in the validation have received precise instructions and are experienced registrars. There are no settings available in Sweden for the automatic transfer of data.

Involving only five units in the calculation of completeness of cases, response rate and control of incomplete and missing data could introduce a selection bias. The selection of units was made to imitate the Swedish system with a mixture of units regarding both sizes and settings and covers 33% of the register entries. However, the willingness to report to the register varies between units and individual physicians, which is why the results could differ locally. To compensate for this, the validation of the data accuracy was made by extracting every 20^th^ registration to ensure a random selection of units.

Statistics from SwedEar are publicly available at the website and can be accessed by the public, authorities and healthcare professionals, which is why this validation of the data quality and knowledge of its limitations are crucial to understand the data and ensure correct use. Information regarding some limitations of the data is available in the informative text at the website. This study clarifies the strengths and limitations of the data in SwedEar, which are especially important to consider in research on the included subgroups.

## Conclusion

SwedEar is a unique national quality register for chronic ear surgery and has high completeness and excellent coverage. The data in the register have an overall almost perfect reliability. This study establishes that SwedEar can be used for monitoring quality aspects and results in myringo- and ossiculoplasty for clinical, research and public purposes. More work is needed to increase the completeness and response rate. Since this study, SwedEar has expanded to include all chronic ear surgeries which reduces uncertainties due to inclusion criteria and might increase inclusion. Due to the addition of new variables, it is not possible to extrapolate all the results to the renewed register and a repeated validation assessment is recommended.

## Data Availability

The use of data from SwedEar is regulated by Swedish law. The data that support the findings of this study are available from the Center of Registers, Västra Götaland, Sweden (registercentrum@vgregion.se), but restrictions apply to the availability of these data, which were used under licence for the current study, and so are not publicly available. Data from SwedEar are however available from the authors (Åsa Bonnard) upon reasonable request and with permission from the Center of Registers, Västra Götaland, Sweden.

## Abbreviations

SwedEar: The Swedish Quality Register for Ear Surgery
QRMO: Quality Register for Myringo- and Ossiculoplasty
PINs: Personal identification numbers
SÖF: The Swedish Otosurgical Society
PROM: Patient-Reported outcome measures
TM: Tympanic membrane
PPV: Positive predictive value
ICC: Interclass correlation coefficient
dB: decibel
PTA4: pure tone average of 0.5, 1, 2, 4 kHz

## References

[1] Carroll JD, Mack MJ, Vemulapalli S, Herrmann HC, Gleason TG, Hanzel G (2020). STS-ACC TVT Registry of transcatheter Aortic valve replacement. J Am Coll Cardiol.

[2] Parry-Jones AR, Paley L, Bray BD, Hoffman AM, James M, Cloud GC (2016). Care-limiting decisions in acute stroke and association with survival: analyses of UK national quality register data. Int J Stroke.

[3] Puechmaille M, Lambert C, Aubry K, Bordure P, Bozorg-Grayeli A, Deguine O (2020). The French National Cochlear Implant Registry (EPIIC): Bilateral cochlear implantation. Eur Ann Otorhinolaryngol Head Neck Dis.

[4] Mitchell D, Venermo M, Mani K, Bjorck M, Troeng T, Debus S (2015). Quality improvement in vascular surgery: the role of comparative audit and vascunet. Eur J Vasc Endovasc Surg.

[5] Smith Jervelund S, De Montgomery CJ (2020). Nordic registry data: value, validity and future. Scand J Public Health.

[6] SKR information kvalitetsregister engelska [Internet]. Available from: https://skr.se/en/kvalitetsregister/omnationellakvalitetsregister.52218.html Accessed 9^th^ Sept 2022.

[7] Pauli N, Finizia C, Lundman L, Björsne A, Dahlin-Redfors Y. Are there differences in revision stapes surgery outcomes between university and county clinics? A study from the quality register for otosclerosis surgery in Sweden. Eur Arch Otorhinolaryngol. 2023;280(5):2247–2255. 10.1007/s00405-022-07737-5. 10.1007/s00405-022-07737-5 PMC10066141 36367582

[8] The Swedish Quality Register for Ear surgery, Homepage and Registration [Internet]. 2022. Available from: https://myr.registercentrum.se/ Accessed 9^th^ Sept 2022.

[9] Young M, Gjuric M, Haeusler R. An international otology database. Otol Neurotol. 2005; 26(5):1087–92. 10.1097/01.mao.0000185045.31276.10 16151364

[10] Arts DG, De Keizer NF, Scheffer GJ. Defining and improving data quality in medical registries: a literature review, case study, and generic framework. J Am Med Inform Assoc. 2002;9(6):600–11. 10.1197/jamia.m1087. 10.1197/jamia.M1087 PMC349377 12386111

[11] Hoeijmakers F, Beck N, Wouters MWJM, Prins HA, Steup WH (2018). National quality registries: how to improve the quality of data?. J Thorac Dis.

[12] SÖF [Internet]. [cited 2022 Mar 21]. Available from: https://www.svenskonh.se/delforeningar/sof/statistik/.

[13] Wongpakaran N, Wongpakaran T, Wedding D, Gwet KL (2013). A comparison of Cohen’s Kappa and Gwet’s AC1 when calculating inter-rater reliability coefficients: a study conducted with personality disorder samples. BMC Med Res Methodol.

[14] Gisev N, Bell JS, Chen TF (2013). Interrater agreement and interrater reliability: Key concepts, approaches, and applications. Res Soc Adm Pharm.

[15] Landis J, Koch G (1977). The measurement of observer agreement for categorical data. Biometrics.

[16] Koo TK, Li MY (2016). A guideline of selecting and reporting intraclass correlation coefficients for reliability research. J Chiropr Med.

[17] Shrout PE, Joseph S. Intraclass correlations: uses in assessing rater reliability psychological bulletin. Psychol Bull. 1979; 86(2):420–8. 10.1037//0033-2909.86.2.420 18839484

[18] Varnum C, Pedersen AB, Gundtoft PH, Overgaard S (2019). The what, when and how of orthopaedic registers: an introduction into register-based research. EFORT Open Rev.

[19] Norman M, Källén K, Wahlström E, Håkansson S, The SNQ Collaboration, Skiöld B, et al. The Swedish Neonatal Quality Register – contents, completeness and validity. Acta Paediatr. 2019;apa.14823. 10.1111/apa.14823 31006126

[20] On behalf of the steering group of the National Register for Breast Cancer, Löfgren L, Eloranta S, Krawiec K, Asterkvist A, Lönnqvist C, et al. Validation of data quality in the Swedish National Register for Breast Cancer. BMC Public Health. 2019;19(1):495. 10.1186/s12889-019-6846-6 PMC6498669 31046737

[21] Govatsmark RES, Janszky I, Slørdahl SA, Ebbing M, Wiseth R, Grenne B (2020). Completeness and correctness of acute myocardial infarction diagnoses in a medical quality register and an administrative health register. Scand J Public Health.

[22] Lundström F, Odhagen E, Alm F, Hemlin C, Nerfeldt P, Sunnergren O (2022). A validation study of data in the National Tonsil Surgery Register in Sweden: high agreement with medical records ensures that data can be used to monitor clinical practices and outcomes. BMC Med Res Methodol.

[23] Wennberg S, Karlsen LA, Stalfors J, Bratt M, Bugten V (2019). Providing quality data in health care - almost perfect inter-rater agreement in the Norwegian tonsil surgery register. BMC Med Res Methodol.

[24] Sundbøll J, Adelborg K, Munch T, Frøslev T, Sørensen HT, Bøtker HE (2016). Positive predictive value of cardiovascular diagnoses in the Danish National Patient Registry: a validation study. BMJ Open.

[25] National Patient Register [Internet]. Available from: https://www.socialstyrelsen.se/en/statistics-and-data/registers/national-patient-register/ Accessed 9^th^ Sept 2022.

[26] Bergdahl C, Nilsson F, Wennergren D, Ekholm C, Möller M (2021). Completeness in the Swedish Fracture Register and the Swedish National Patient Register: an assessment of Humeral Fracture Registrations. Clin Epidemiol.

[27] SKR certification levels [Internet]. [cited 2022 Mar 21]. Available from: https://skr.se/kvalitetsregister/omnationellakvalitetsregister/qualityregistries/findaregistry/certificationlevels.54725.html.

[28] Nahm ML, Pieper CF, Cunningham MM (2008). Quantifying data quality for clinical trials using electronic data capture. PLoS One.

[29] Kirch W. Database Error Rate. In: Kirch W. (eds) Encyclopedia of Public Health. Dordrecht: Springer;2008. 10.1007/978-1-4020-5614-7_667.

[30] Berglund M, Florentzson R, Fransson M, Hultcrantz M, Eriksson PO, Englund E (2017). Myringoplasty Outcomes From the Swedish National Quality Registry: Myringoplasty Outcomes in a Swedish Database. Laryngoscope.

[31] Phillips JS, Yung MW, Nunney I (2015). Myringoplasty outcomes in the UK. J Laryngol Otol.

[32] Karela M, Berry S, Watkins A, Phillipps JJ (2008). Myringoplasty: surgical outcomes and hearing improvement: is it worth performing to improve hearing?. Eur Arch Otorhinolaryngol.

[33] Lee P, Kelly G, Mills RP (2002). Myringoplasty: does the size of the perforation matter?. Clin Otolaryngol Allied Sci.

